# Ruptured Hepatic Hemangioma in the Third Trimester of Pregnancy: A Rare Case Report

**DOI:** 10.7759/cureus.25397

**Published:** 2022-05-27

**Authors:** Haven Ward, Omid Hosseini, Yohey Hashimoto, Basem Soliman

**Affiliations:** 1 School of Medicine, Texas Tech University Health Sciences Center, Lubbock, USA; 2 School of Medicine, Texas Tech University Health Sciences Center, Amarillo, USA; 3 Department of Surgery, Texas Tech University Health Sciences Center, Amarillo, USA

**Keywords:** hemorrhage, surgical management, giant hemangioma, hemangioma, ruptured hepatic hemangioma

## Abstract

Hepatic hemangiomas are considered the most common benign mesenchymal hepatic tumors. Most cases are asymptomatic. However, giant hemangiomas can present with a variety of clinical presentations, with a rupture being the most catastrophic outcome. Only a few cases of ruptured perinatal hepatic hemangiomas have been reported. Accelerated growth of hepatic hemangiomas caused by increased estrogen in pregnancy, increased intra-abdominal pressure, and direct contact with a gravid uterus are possible mechanisms for increased risk of rupture during pregnancy. The safety of either non-operative or surgical treatment of symptomatic giant hemangioma during pregnancy has not been adequately investigated. We present a rare case of a 28-year-old G1P0 female at 33 weeks gestation that presented with a ruptured hepatic hemangioma treated with damage control surgery followed by nonanatomic surgical resection.

## Introduction

Hepatic hemangiomas are considered the most common benign mesenchymal hepatic tumors, with rare malignant transformation [1]. The size can range from a few millimeters to over 20 cm, those over 5 cm are considered giant hemangiomas [2]. Most cases are asymptomatic. Giant hemangiomas can present with a variety of clinical presentations with rupture being the most catastrophic outcome and is the reason why correct diagnosis and management are important [2]. The mortality rate of a ruptured hemangioma has been reported to be as high as 75% [3]. Treatment for symptomatic hepatic hemangiomas should be based on the size and location of the tumor [3]. Relative surgical indications are spontaneous or traumatic rupture with hemoperitoneum, intratumoral bleeding, and consumptive coagulopathy known as Kassabach-Merrit syndrome [3]. The proposed treatment options for ruptured hepatic hemangiomas are anatomic/nonanatomic resection, transcatheter hepatic arterial embolization (TAE), and liver transplant [4]. Due to the paucity of literature, the safety of either non-operative management or surgical treatment of symptomatic giant hemangioma during pregnancy has not been adequately investigated [5]. We present a rare case of a 28-year-old G1P0 female at 33 weeks gestation that presented with a ruptured hepatic hemangioma treated with damage control surgery followed by nonanatomic surgical resection.

## Case presentation

A previously healthy 28-year-old G1P0 woman at 33 weeks gestation presented to the emergency department (ED) complaining of right upper quadrant pain, nausea, vomiting, and hypertension. The patient was seen in a clinic previously and thought to have cholelithiasis. Records from the clinic indicated elevated liver function tests (LFTs), stable hemoglobin, and normal antenatal ultrasonograms. Blood pressure at prenatal visits was 154/102 mmHg at 26 weeks and 161/83 mmHg at 30 weeks. At the ED the patient endorsed constant right upper quadrant pain that radiated to her lower abdomen and right shoulder that worsened with palpation. Her blood pressure was 175/95 mmHg. Ultrasound showed no evidence of cholelithiasis or acute cholecystitis. A 5.3 cm × 11.7 cm × 5.5 cm isoechoic liver mass was visualized and appeared to have active bleeding concerning a ruptured hepatic hemangioma. Fetal heart rate tracing was noted as a normal category 1. At this time, the patient was taken emergently to the operating room as cesarean delivery was indicated due to recurrent late and variable decelerations and for maternal status with concerns for active intrabdominal bleeding. Maternal labs obtained at the time of admission were as followed, hemoglobin 12.6 g/dL, hematocrit 34%, platelets 116 x 103/ fibrinogen 623 mg/dL, creatinine 0.5 mg/dL, aspartate aminotransferase (AST) 275 units/L, and alanine aminotransferase (ALT) 236 units/L.

The delivery of a 33-week infant was successful through a cesarean section. General surgery was called due to intraperitoneal bleeding secondary to the liver mass. Through a Pfannenstiel incision, the liver mass was able to be palpated with a large amount of clotted blood and 200-300 cc of fresh blood. The decision was made to perform an upper midline incision to evaluate and control the liver hemorrhage. The dome of the liver was noted to have a large, ruptured hemangioma with active bleeding from the inferior edge. Initial attempts to stop the bleeding with pressure/cautery failed. Hepatorrhaphy with a fibrin sealant patch was placed over the ruptured subscapular hematoma. Given the patient's hemodynamic instability at that time, the liver was packed with three large laparotomy pads. A temporary abdominal closure system with negative pressure therapy was placed with a plan to return for definitive treatment after stabilization. OB/GYN then closed the cesarean section. The patient was transferred to the surgical intensive care unit (SICU), intubated, and in guarded condition.

**Figure 1:**
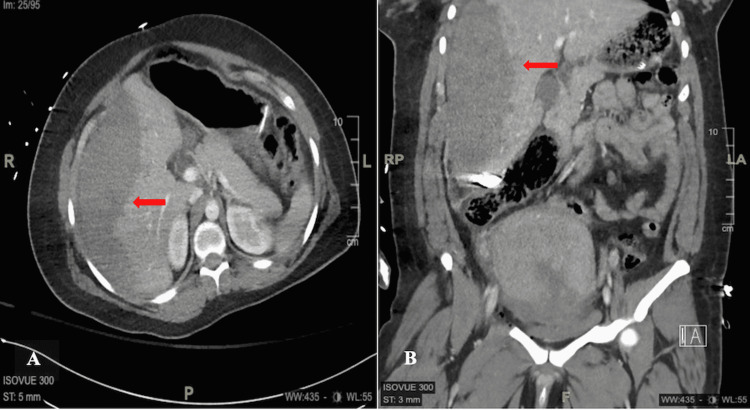
(A) Axial and (B) coronal views: liver subcapsular hematoma measuring up to 5 cm in thickness with mass effect causing a rightward shift of the liver, compression of the right kidney, and the intrahepatic IVC

Postoperative computerized tomography (CT) with intravenous contrast was conducted to determine if there was any current active hemorrhage. CT did not show any evidence of active intra-abdominal hemorrhage. It did show a large liver subcapsular hematoma measuring up to 5 cm in thickness and mass effect causing a rightward shift of the liver, compression of the right kidney, and the intrahepatic inferior vena cava (Figures 1A, 1B).

**Figure 2:**
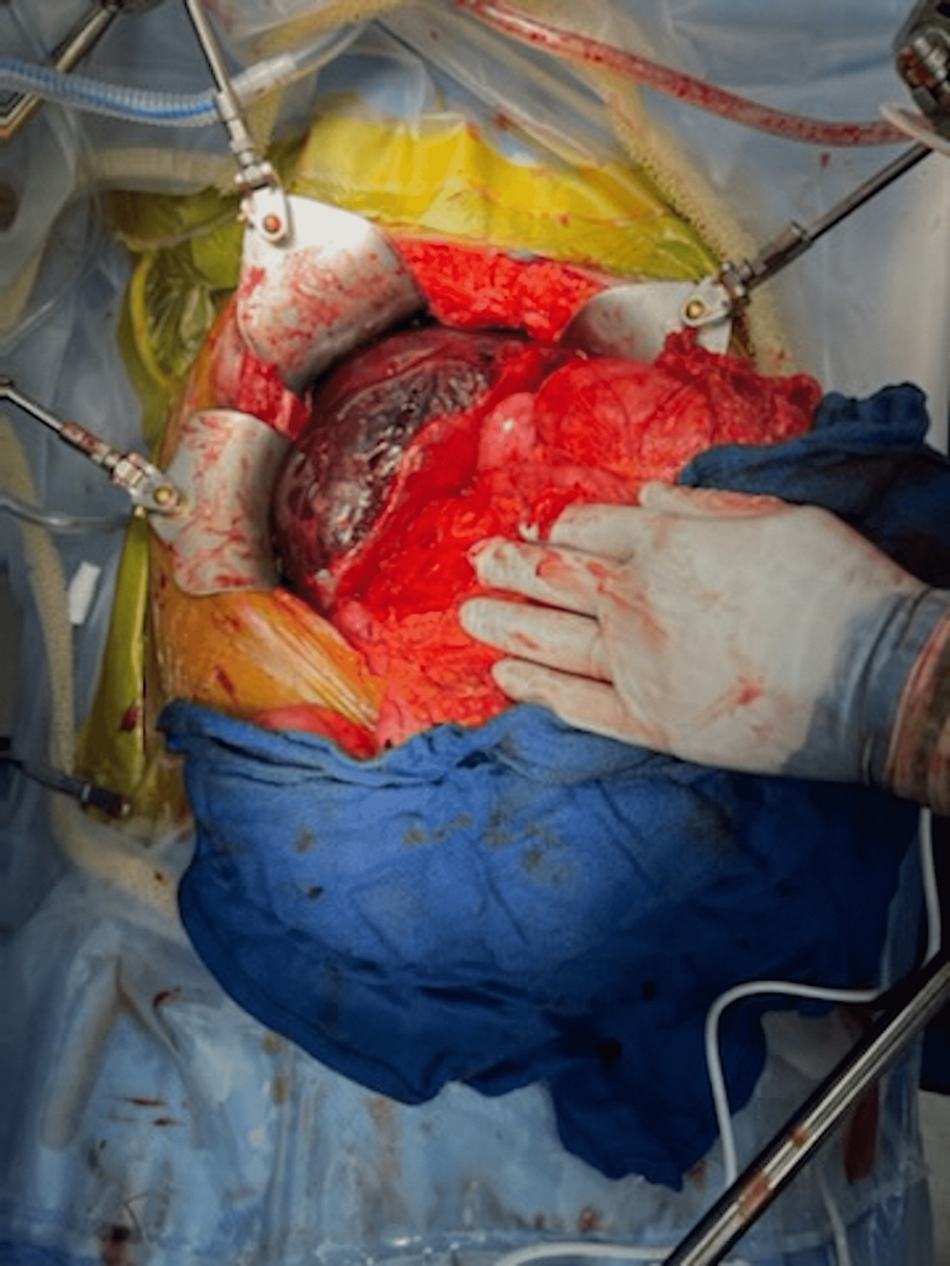
The intraoperative initial view of the inferior subscapular hepatic hematoma

Postoperatively, the patient was kept intubated. The laboratory studies showed a slow decrease in hemoglobin level that required two units of packed red blood cell transfusions and significantly elevated AST and ALT of 2,378 units/L and 2,234 units/L, respectively. Four days later, the patient's hemodynamics stabilized and was taken back to the operating room for open exploratory laparotomy and possible hepatic resection. Initial exploration showed an apparent hepatic hematoma involving segments five, six, and seven (Figure 2).

**Figure 3:**
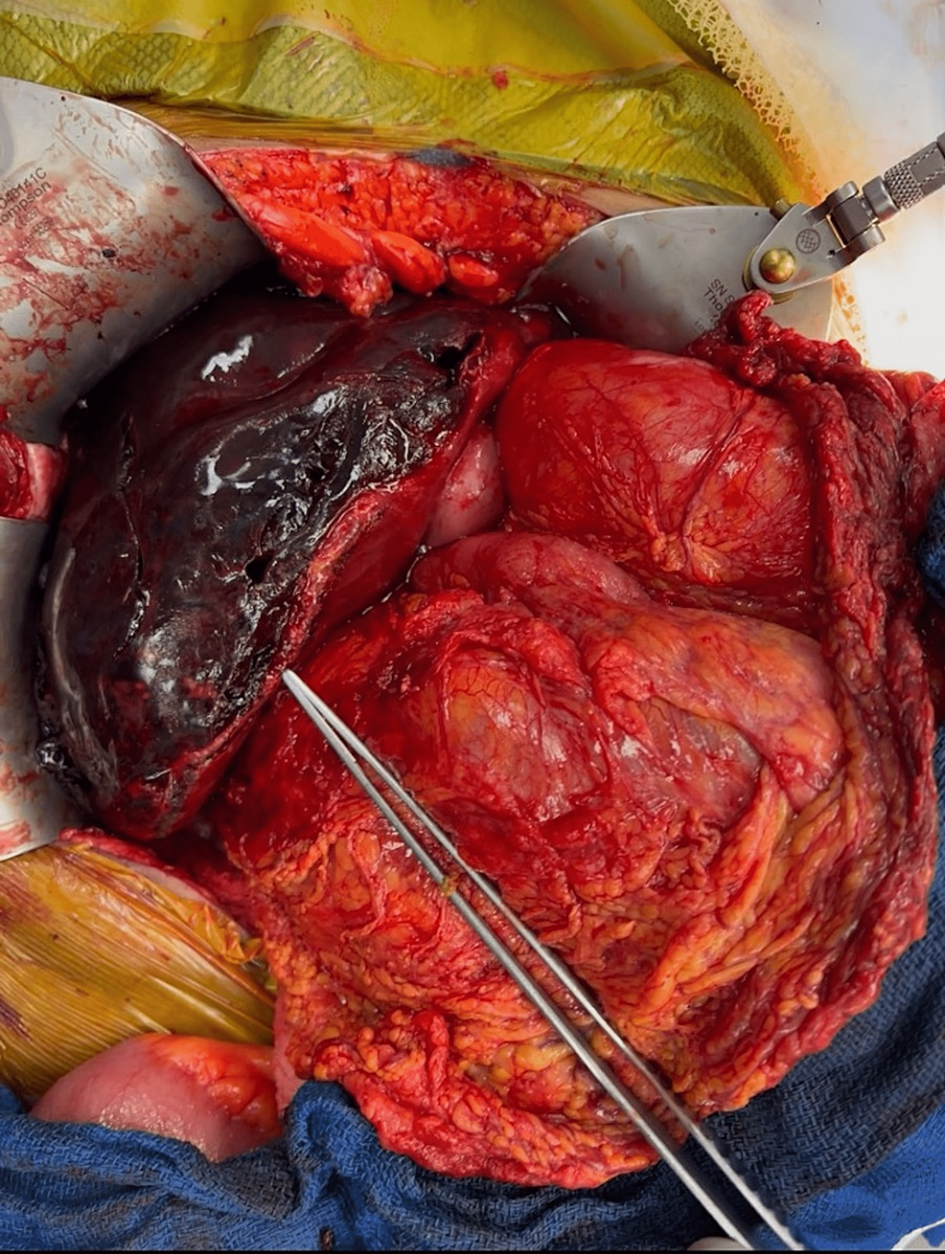
Intraoperative view of the mobilized right lobe of the liver with hematoma involving segments five, six, and seven

The right lobe of the liver was adequately mobilized allowing full visualization and manipulation of the tumor (Figure 3).

Using monopolar electrocautery and a bipolar energy device, the liver parenchyma in segments five, six, and seven were dissected to include the hepatic hematoma. The resected margins were hemostatic and covered with an omental patch. Cholecystectomy was performed as future cholecystectomy would be difficult. The patient tolerated the product well and was transferred back to the SICU. At the two-week follow-up visit, the patient presented in good condition without complications.

## Discussion

In the case of hepatic hemangioma rupture during pregnancy, it should be noted that the presentation of symptoms can be easily misdiagnosed for other less morbid conditions and that management of the patient should be individualized based on the condition of the fetus and mother [6]. Maternal mortality of subscapular liver hematoma ranges from 17% to 59% depending on rupture, the timing of diagnosis, and therapeutic interventions [7]. The perinatal mortality rate has been reported as high as 42% [7]. A study reported by Schnaper et al. demonstrated that estrogen enhances endothelial cell proliferation, migration, and organization into capillary-like structures which leads to increased angiogenesis [8]. For this reason, accelerated growth caused by increased estrogen in pregnancy, as well as increased intra-abdominal pressure, and direct contact with a gravid uterus are possible mechanisms for increased risk of rupture during pregnancy [5,9].

Few cases regarding rupture of hepatic hemangioma during pregnancy have been reported. Ryu et al. reported a case of a 36-year-old female G1P0 at 34 weeks gestation with twin pregnancy presented with chest pain and hemodynamic instability. The patient underwent an emergent cesarean section under the impression of possible pulmonary embolism with incidental finding of a large hepatic hematoma. The patient underwent hepatic angiography that demonstrated an 8 cm subscapular hematoma [6]. Selective catheterization and embolization of the left hepatic artery were performed on postoperative day 2 without complication [6]. Another case reported by Tegen et al. demonstrated successful management of a ruptured subcapsular hematoma diagnosed intra-operatively with liver packing with hemostatic agents and a drainage catheter placement [10]. Furthermore, Holst et al. reported a diagnosis of a ruptured liver hemangioma that was made postoperatively in a case of a 33-year-old G2P1 when continued hemorrhage was noted [11]. The patient required an emergent exploratory laparotomy for hematoma evacuation, which revealed a large ruptured subcapsular liver hematoma that was controlled with local surgical hemostasis. A postoperative CT scan noted multiple cavernous hemangiomas with no active bleeding and selective embolization was not possible due to the bilateral nature of hemangiomas. The patient was taken to the intensive care unit and received multiple transfusions and a continuous infusion of aprotinin. She remained stable and extubated five days later without suffering from complications after six months follow-up [11].

Our patient’s presentation suggests multiple critical features of the hepatic hemangioma in pregnancy. Presentation of symptoms can be vague; diagnosis may be difficult due to the gravid uterus and the limitations of radiation exposure. For these patients, definitive hepatic resection can be used as a successful method of treatment.

## Conclusions

The mechanism behind the natural course and risk factors for rupture of hepatic hemangiomas is incompletely understood. In a pregnant patient presenting with acute abdominal pain of unknown cause, increased LFTs, and hemodynamic instability, ruptured hepatic hemangioma should be considered one of the differential diagnoses. The presence of ruptured subcapsular liver hematoma that results in shock is a surgical emergency. Therefore, a multidisciplinary approach involving surgical, interventional radiology, and obstetric specialists should be used for the diagnosis and treatment planning of symptomatic hepatic hemangiomas.
